# Dock4 contributes to neuropathic pain by regulating spinal synaptic plasticity in mice

**DOI:** 10.3389/fnmol.2024.1417567

**Published:** 2024-08-30

**Authors:** Qiaochu Fu, Hongyi Li, Zhuanxu Zhu, Wencui Li, Zhihua Ruan, Ruijie Chang, Huixia Wei, Xueqin Xu, Xunliang Xu, Yanqiong Wu

**Affiliations:** ^1^Department of Anesthesiology, Beijing Tiantan Hospital, Capital Medical University, Beijing, China; ^2^Department of Anesthesiology, National Cancer Center/National Clinical Research Center for Cancer/Cancer Hospital, Chinese Academy of Medical Sciences and Peking Union Medical College, Beijing, China; ^3^Department of Gynaecology, Taihe Hospital, Hubei University of Medicine, Hubei, China; ^4^Institute of Anesthesiology & Pain (IAP), Department of Anesthesiology, Department of Gynecology, Taihe Hospital, Hubei University of Medicine, Hubei, China; ^5^Department of Anesthesiology and Pain Medicine, Hubei Key Laboratory of Geriatric Anesthesia and Perioperative Brain Health, Wuhan Clinical Research Center for Geriatric Anesthesia, Tongji Hospital, Tongji Medical College, Huazhong University of Science and Technology, Wuhan, China

**Keywords:** DOCK4, NMDA, neuropathic pain, synaptic plasticity, spinal cord

## Abstract

**Introduction:**

Neuropathic pain (NP) conditions arising from injuries to the nervous system due to trauma, disease, or neurotoxins are chronic, severe, debilitating, and exceedingly difficult to treat. However, the mechanisms of NP are not yet clear. Here we explored the role of Dock4, an atypical Rac1 GEF, in the development of NP.

**Methods:**

Mechanical allodynia was assessed as paw withdrawal threshold by a dynamic plantar aesthesiometer. Immunofluorescence staining was conducted to investigate the expression and localization of Dock4, Rac1 and GluN2B. Quantitative analysis of Dock4, Rac1 and GluN2B were determined by qRT-PCR and Western blot assay. Spontaneous excitatory and inhibitory postsynaptic currents in spinal cord slices were examined using whole cell patch clam. Dendritic spine remodeling and synaptogenesis were detected in cultured dorsal spinal neurons.

**Results and discussion:**

We found that SNL caused markedly mechanical allodynia accompanied by increase of Dock4, GTP-Rac1and GluN2B, which was prevented by knockdown of Dock4. Electrophysiological tests showed that SNL facilitated excitatory synaptic transmission, however, this was also inhibited by Dock RNAi-LV. Moreover, knockdown of Dock4 prevented dendritic growth and synaptogenesis.

**Conclusion:**

In summary, our data indicated that Dock4 facilitated excitatory synaptic transmission by promoting the expression of GluN2B at the synaptic site and synaptogenesis, leading to the occurrence of NP.

## 1 Introduction

Neuropathic pain (NP) is a chronic pain condition resulting from injuries to the nervous system caused by trauma, infection, or illness. In recent years, the global incidence of NP has been continuously rising, affecting 7–10% of the world’s population and becoming a significant and challenging medical issue (Bouhassira, 2019; Bannister et al., 2020). Its clinical manifestations include uncomfortable sensations such as stabbing or burning pain, numbness, allodynia. Previous studies have reported synaptic plasticity as a crucial mechanism in the development of NP. However, the molecular mechanisms underlying synaptic plasticity in NP remain unclear.

Synaptic plasticity encompasses changes in the structure and function of synapses, leading to improved synaptic transmission efficiency (Haas et al., 2018; Dai et al., 2022). Receptor-mediated postsynaptic activity and alterations in neurotransmitters release from the presynaptic terminal are pivotal in synaptic plasticity formation (Franchini et al., 2020; Li et al., 2023). The N-methyl-D-aspartate receptor (NMDAR) is a key player in the glutamate excitatory system. GluN2B, a subunit of the ionotropic glutamate NMDAR, modulates synaptic plasticity and facilitates central sensitization (Chen et al., 2021; Zhang et al., 2022). Growing evidence indicates that GluN2B are involved in nociceptive transmission and pain regulation in the central nervous system (CNS), playing a critical role in the development of chronic pain (Chia et al., 2020; Meng and Shen, 2022). Although the GluN2B subunit plays a unique role in nociceptive signaling, further studies are required to determine the precise molecular mechanisms by which the GluN2B facilitates the development and hypersensitivity of NP.

Recent studies showed that NMDAR could be regulated by Rac1 (Tolias et al., 2005; Xiao et al., 2013). Shank3-deficient mice display reduced NMDAR synaptic function and distribution, which is correlated with blunted Rac1 activity. The activity of Rac1 is intricately regulated by Rac1 guanine nucleotide exchange factors (GEFs; Guo et al., 2021). Dock4, a member of the Dock (dedicator of cytokinesis) family, an unconventional Rac1 GEF associated with ASD and other neuropsychiatric conditions (Guo et al., 2021). Furthermore, Dock4 has been shown to play critical roles in axon guidance, dendritic development, and dendritic spine formation (Ueda et al., 2008, 2013; Huang et al., 2019). Studies have revealed that Dock4 deficiency significantly diminishes Rac1 activity in the hippocampus and reduces the expression of NMDAR subunits. The neural communication mediated by GluN2B was also decreased in the hippocampus in Dock4 knockout mice. Restoring the function of Rac1 can reverse Dock4 knockdown-mediated reduction of GluN2B. However, it remains unclear whether Dock4 regulates GluN2B to influence synaptic transmission and contribute to the development of NP.

To explore the role of Dock4 in NP, a spinal nerve ligation (SNL) murine model was established to assess the NP behaviors. Dock4 RNAi-LV was administered to decrease the mRNA and protein levels of Dock4. Whole-cell patch clamp recording was performed to evaluate changes in synaptic transmission. The findings indicated that silencing Dock4 reversed NP behaviors and suppressed excitatory synaptic transmission, GluN2B expression, and synaptogenesis. These results suggest that Dock4 contributes to neuropathic pain by modulating spinal synaptic plasticity in mice.

## 2 Materials and methods

### 2.1 Animals

Male or female C57 mice weighing approximately 18–25 g were obtained from the Animal Center of Hubei University of Medicine. The mice were given free access to water and food and were housed in a 12-h light–dark cycle. All animal experiments were approved by the Animal Use and Care Committee of Hubei University of Medicine.

### 2.2 Lentivirus construction and microinjection

Microinjection of lentivirus into the spinal dorsal horn (SDH) was performed as previously described (Ke et al., 2017). For the Dock4 RNAi experiment, mouse Dock4 scramble (5′-GTGCATTGTACTGGTCTTT-3′) or shRNA (5′-GAAGTTGTTCGGTTTCTCT-3′) were designed according to the literatures (Bai et al., 2018). Lentivirus vector construction and production were completed by Shanghai Gene Chem Co. Ltd., China. Briefly, following isoflurane inhalation, the mice were positioned prone on the operating table. A longitudinal incision was made at the midline of the lower lumbar region, and the laminae were removed to expose the L4-6 spinal cord. A glass capillary was inserted 0.4 mm to the left of the midline of the SDH at a depth of 300 μm. Subsequently, shRNA solutions were injected at a rate of 0.4 μl/min into the two sites (approximately 400–500 nL in one site) (Kohro et al., 2020; Zhang et al., 2024). The glass capillary was left in place for 2 min after microinjection. After suturing the wound layer by layer, the mice were placed on a rewarming blanket for recovery and then returned to the cage. Mice were allowed to recover for at least 2 weeks after injection of lentiviral before the NP experiments.

### 2.3 Establishment of NP model

The SNL (Spinal Nerve Ligation) procedure was conducted following the method described in previous studies (Cai et al., 2005; Gomez et al., 2024). After inducing anesthesia with isoflurane, a 1 cm longitudinal incision was made approximately 5 mm lateral to the left of the midline, with 2/3 of the incision positioned on the iliac crest. The thoracolumbar fascia was incised, and the erector spinae muscle was gently separated. The L6 transverse process was excised to expose the L5 spinal nerve under a surgical microscope. Subsequently, a No.6.0 silk thread was utilized to securely ligate the L5 spinal nerve. In the sham-operated control group, the same procedure was followed, but the spinal nerve was not ligated.

### 2.4 Behavioral tests

The mice were allowed to acclimate in plexiglass boxes with an elevated metal mesh floor for 15–20 min before the behavioral tests. The paw withdrawal threshold (PWT) in response to mechanical stimulation was assessed using a dynamic plantar aesthesiometer (Ugo Basile, Stoelting, IL, USA). A steel rod was applied against the hind paw with ascending force from 0 to 10 g over a 10-s period. The mechanical stimulus ceased automatically upon paw withdrawal, and the threshold was determined as an average. Each mouse underwent 4 measurements at 5- min intervals.

### 2.5 Culture of mouse dorsal spinal neurons

Primary cell cultures of mouse dorsal spinal neurons were prepared following the methodology outlined in a previous study (Cao et al., 2017). A pregnant C57BL/6 mouse was euthanized via cervical dislocation after inhalation anesthesia. Embryonic day 14 (E14) embryos were dissected, and the dorsal half of spinal cords were isolated after making a dorsal incision in the embryos. The dissected tissues were washed, minced with scissors, and then digested with 1% trypsin solution for 10 min at 37°C. The digested tissue fragments were centrifuged (1,000 *g* × 5 min) and filtered to obtain a cell suspension. This cell suspension was seeded onto polyethyleneimine-coated glass coverslips in basal culture medium with 2% B27 supplement (Invitrogen) and 0.5 mM glutamine. The cultured cells were then placed in an incubator at 37°C with 5% CO_2._ The Dock4 RNAi-Lentivirus (RNAi-LV) or negative control lentivirus (NC-LV) was introduced into culture medium on days *in vitro* (DIV) 10 and evaluated on DIV 15.

### 2.6 Quantitative RT-PCR

Total RNA from the L4-L6 spinal cord was isolated using the RNeasy Mini Kit (Invitrogen, USA). Real-time PCR amplifications were conducted using the QuantStudio™3 Real-Time PCR System (Thermo Fisher Scientific Inc., Waltham, MA, USA). Each PCR reaction was performed in triplicate, and the GAPDH gene was utilized as an internal standard for normalization. The primers sequences used for RT-PCR are provided below: GAPDH sense: 5′-AGGTCGGTGTGAACGGATTTG-3′, antisense: 5′-TGTAGACCATGTAGTTGAGGTCA-3′; GluN2B sense: 5′-TGGTTTCAGGCTGGTAGAGG-3′, antisense: 5′-AGCACCTTCCTAATGGCAGT-3′; Dock4 sense: 5′-TGGGGAACCATGTGGAAACAG-3′, antisense: CGTCGCAGATCGAGGATTTCA. These primers were used for the real-time PCR analysis to quantify the expression levels of the target genes in the L4-L6 spinal cord samples.

### 2.7 Electrophysiological recordings

Spinal cord slice preparation and patch-clamp recordings were performed as described previously (Wu et al., 2022). Mice were anesthetized with isoflurane, and the lumbar spinal cord (L4–L6) were rapidly removed and immersed in ice-cold artificial cerebrospinal fluid containing (in mM) 234 sucrose, 3.6 KCl, 1.2 MgCl2, 2.5 CaCl2, 1.2 NaH2PO4, 12.0 glucose, and 25.0 NaHCO3, pre-saturated with 95% O2 and 5% CO_2_. Transverse slices (400 μm thickness) were prepared using a VT-1000S vibratome (Leica, Germany) and then kept in an incubation chamber that contains artificial cerebrospinal fluid ACSF (in mM): 117.0 NaCl, 3.6 KCl, 1.2 MgCl_2_, 2.5 CaCl_2_, 1.2 NaH_2_PO_4_, 11.0 glucose, and 25.0 NaHCO_3_, equilibrated at 32°C with 95% O_2_–5% CO_2_. After the incubation for 1 h, the slices were moved to a recording chamber and perfused with ACSF (32°C) saturated with mixed gas at a flow rate of 2 mL per minute while submerged.

Neurons located in the second lamina of the spinal cord were chosen for electrophysiological recording due to their primary reception of pain-related signals from sensory nerve fibers (Pan and Pan, 2004; Santos et al., 2007; Chen et al., 2014). Lamina II neurons were visualized under an upright microscope (Olympus). Whole-cell recordings were made with MultiClamp 700B amplifier and 1440A digitizer (Molecular Devices). For the recording of sEPSC, 100 μM picrotoxin was added to the perfusion solution to block GABA_A_ receptor-mediated postsynaptic responses. For the recording of sIPSC, 50 μM APV and 5 μM CNQX were added to the perfusion solution to block NMDA and AMPA receptor-mediated postsynaptic responses. To minimize the Mg^2+^ block of NMDARs, NMDA currents were recorded in an extracellular solution containing no Mg^2+^ at a holding potential of −60 mV (Chen et al., 2018; Li et al., 2021). The pipette internal solution contained (in mM) 110.0 Cs_2_SO_4_, 2.0 MgCl_2_, 0.1 CaCl_2_, 1.1 EGTA, 10.0 HEPES, 2.0 Mg-ATP and 0.3 Na_2_GTP (pH was adjusted to 7.25 with 1.0 M CsOH; 280–300 mOsmol/L). Neurons were held at membrane potentials of −60 and 0 mV, respectively, for the purpose of recording spontaneous excitatory postsynaptic currents (sEPSCs) and inhibitory postsynaptic currents (IPSCs). All these items were measured by Clampfit software (Axon Instruments, Foster City, CA).

EPSCs mediated by AMPARs were recorded at −60 mV in the presence of APV (100 μM) and PTX (50 μM), while EPSCs mediated by NMDARs were recorded at +40 mV in the presence of CNQX (20 μM) and PTX (50 μM). These currents were elicited through electrical stimulation (0.6 mA, 0.5 ms) of the ipsilateral dorsal root using a tungsten bipolar electrode. Inter-stimulus intervals longer than 15 s were utilized to reduce depression resulting from repetitive stimulation, and at least 10 responses were averaged for each intensity to quantify the AMPAR-EPSCs and NMDAR-EPSCs. To calculate the NMDAR-to-AMPAR EPSC ratio, AMPAR-mediated EPSCs were initially recorded in an artificial cerebrospinal fluid (ACSF) solution (containing PTX) at −60 mV. Subsequently, the same cell was maintained at +40 mV to record NMDAR-mediated EPSCs in the presence of CNQX (10 μM) and PTX (50 μM) using the same stimulus pulse. The ratio of the maximal amplitude of NMDAR-EPSCs to AMPAR-EPSCs was defined as the NMDAR/AMPAR ratio. To evaluate changes in the functional proportion of NR2B subunits at synapses from SNL + RNAi-LV or SNL + NC-LV mice, the NR2B antagonist ifenprodil (3 μM) was administered via a patch pipette (1.5 μm diameter) using a picospritzer apparatus (General Valve, USA). The proportion of isolated NMDAR current was determined 20–30 min after drug application.

### 2.8 Immunofluorescence

Mice were anesthetized with isoflurane and intracardially perfused with of 20 ml phosphate-buffered solution (PBS) followed by 4% paraformaldehyde to preserve tissue integrity. After perfusion, the L4-L6 spinal segments were carefully extracted and post-fixed overnight. Subsequently, the tissues underwent dehydration in 30% sucrose at 4°C for 48 h. Transverse 30 μm sections of spinal cord were obtained using a cryostat. These sections were then washed three times in PBS for 5 min and treated with 0.3% TritonX-100 for 15 min to permeabilize the membranes. Next, the sections were blocked using 5% bovine serum albumin for 1 h at room temperature. To reduce non-specific binding, a mouse-on-mouse blocking reagent (Jackson Immuno Research Laboratories, AffiniPure Fab Fragment Donkey Anti-Mouse IgG, 715-007-003) was employed. Primary antibodies used in the immunostaining process included: rabbit anti-Dock4 (ab85723,1:100 dilution, Abcam, UK), chicken anti-MAP2 (ab5392, 1:100 dilution; Abcam, UK), mouse anti-Rac1 (05-389, 1,100 dilution, Millipore, USA), guinea pig anti-GluN-2B (244,115, 1,100 dilution, Synaptic Systems, Germany), rabbit anti-synapsin-1 (ab254349, 1:100 dilution, Abcam, UK), rat anti-NeuN (ab279297, 1:200 dilution, Abcam, UK). Following primary antibody incubation, sections were washed thrice with PBS and then incubated with secondary antibodies for 2 h at room temperature. The secondary antibodies used were as follows: Alexa Fluor 488 (goat anti-guinea pig, ab150185, 1:400; Abcam, UK), Alexa Fluor 647 (goat anti-chicken, ab150175, 1:200; goat anti-mouse, ab150115, 1:200; goat anti-rat, ab150167, 1:200; Abcam, UK), Alexa Fluor 594 (goat anti-rabbit, ab150080, 1:200; Abcam, UK), Alexa Fluor™-633 (goat anti-mouse IgG(g1), 1:300; A21126, ThermoFisher). Images were visualized and captured using a confocal microscope (Leica Sp8, Wetzlar, Germany) for further analysis and investigation.

### 2.9 Western blot assay

L4-6 spinal cord tissue and primary mouse dorsal spinal neurons cultures were homogenized on ice for 30 min in radioimmunoprecipitation assay (RIPA) lysis buffer containing phenylmethylsulfonyl fluoride (PMSF), a protease inhibitor (Millipore, USA). Following homogenization, the samples were centrifuged at 12,000 rpm for 15 min at 4°C, and the resulting supernatant was collected. The protein concentration in the supernatant was determined using a BCA kit. The appropriate concentration of SDS-PAGE was prepared according to molecular weight. The separate proteins were then transferred onto a PVDF membrane (Millipore, USA). Membranes were blocked with 5% bovine serum albumin for 2 h at room temperature and probed with antibodies against Dock4 (ab85723, 1:1000 dilution, Abcam, UK), GluN-2B (244,115, 1,500 dilution, Synaptic Systems, Germany), Rac1 (05-389, 1,2000 dilution, Millipore, USA), glyceraldehyde 3-phosphate dehydrogenase (GAPDH, 1:5000, ab128915, Abcam, UK). Next, the membranes were incubated with horseradish peroxidase (HRP)-conjugated secondary antibody for 2 h at room temperatureand visualized using Clarity Western ECL Substrate (Bio-Rad). Blots were quantified using ImageJ software.

The levels of GTP-Rac1 were evaluated by GST pull-down experiments via a Rac1/Cdc42 Activation Assay Kit (17-441, Merck Millipore). According to manufacturer’s instruction, lumbar spinal cord was homogenized with a Mg^2+^ Lysis/Wash Buffer containing 125 mM HEPES (pH 7.5), 750 mM NaCl, 5% Igepal CA-630, 50 mM MgCl_2_, 5 mM EDTA, 10% glycerol and protease inhibitors. Lysates were incubated with agarose beads conjugated with the p21 Rac/Cdc42 binding domain fused to GST (GST-PBD), which specifically binds to GTP-bound Rac1, at 4°C for 60 min. The beads were washed three times with the Mg^2+^ Lysis/Wash Buffer. Bound Rac1-GTP proteins were then resuspended with sample buffer and subjected to Western blot analysis.

### 2.10 Statistical analysis

Data are depicted as means ± standard error of the mean (SEM). Statistical analysis was executed utilizing Prism GraphPad 7.0. Comparative analyses among groups over time were conducted using a two-way repeated-measures (ANOVA), succeeded by Bonferroni’s post-hoc test for pairwise comparisons. For immunofluorescence, qPCR, and western blotting data, intergroup differences were assessed via one-way ANOVA, complemented by Tukey post-hoc test for multiple comparisons. Statistical significance was established at a *p*-value threshold of less than 0.05.

## 3 Results

### 3.1 SNL induces upregulation of Dock4 in the spinal dorsal horn of mice

**Figure 1:**
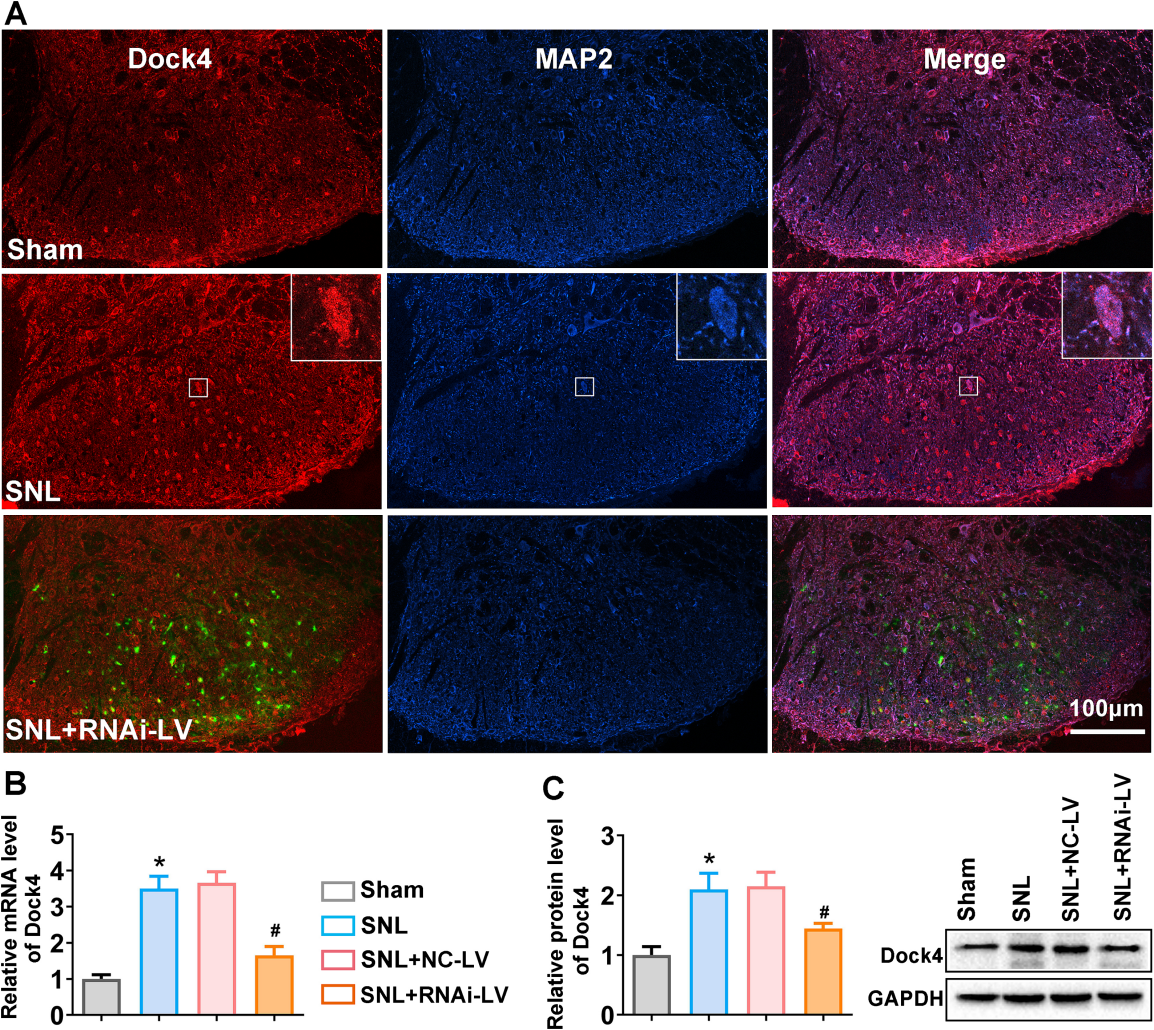
The expression changes of Dock4 in spinal dorsal horn after SNL. **(A)** Double labeling of Dock4 (red) with MAP2 (blue). Green fluorescence represents the lentivirus-transfected neuron and indicates the precise dorsal horn injection site of the virus. The merged images show the location of Dock4 in dorsal horn neurons and indicate upregulation of Dock4 post-SNL which was reversed by RNAi-LV treatment. White rectangles indicated high magnified cell sample of Dock4 and MAP2 colocalization. Scale bar = 100 μm. **(B)** The expression levels of Dock4 mRNA in the lumber spinal cord of sham, SNL, NC-LV, and RNAi-LV group. *p < 0.05, vs. the sham group, *n* = 5; #*p* < 0.05, vs. the NC-LV group, *n* = 5, based on one-way ANOVA, followed by the *post hoc* Tukey test. **(C)** The expression levels of Dock4 protein in the lumber spinal cord of sham, SNL, NC-LV, and RNAi-LV group. A sample western blot is shown on the right. *p < 0.05, vs. the sham group, *n* = 5; #*p* < 0.05, vs. the NC-LV group, *n* = 5, based on one-way ANOVA, followed by the *post hoc* Tukey test.

Initially, we delineated the localization and distribution pattern of Dock4 within the spinal dorsal horn of mice. As depicted in Figure 1A, Dock4 exhibited exclusive colocalization with neuronal elements, as evidenced by its overlapping expression with microtubule-associated protein 2 (MAP2), a canonical neuronal marker. Subsequently, we scrutinized the temporal dynamics of Dock4 mRNA and protein expression in the lumbar region of the spinal dorsal horn in SNL mice. Quantitative qPCR outcomes revealed a pronounced elevation in Dock4 mRNA levels on day 14 post-SNL (*p < 0.05 relative to the sham group). However, this upsurge was notably abrogated by the prophylactic administration of Dock4 RNAi-LV (#p < 0.05 in comparison to the NC-LV; Figure 1B). Corroborating the transcriptomic findings, Western blot analysis demonstrated a substantial augmentation in Dock4 protein levels 14 days subsequent to SNL surgery, when contrasted with the sham-operated cohort (*p < 0.05). Notably, the application of Dock4 RNAi-LV, as opposed to NC-LV, effectively curtailed the SNL-induced upregulation of Dock4 protein (#*p* < 0.05; Figure 1C). Collectively, these data underscore that SNL triggers the activation of Dock4 within dorsal horn neurons, thereby implicating Dock4 as a crucial mediator in the pathogenesis of NP.

### 3.2 Spinal microinjection of Dock4 RNAi-LV reduced SNL-induced pain hypersensitivity

**Figure 2:**
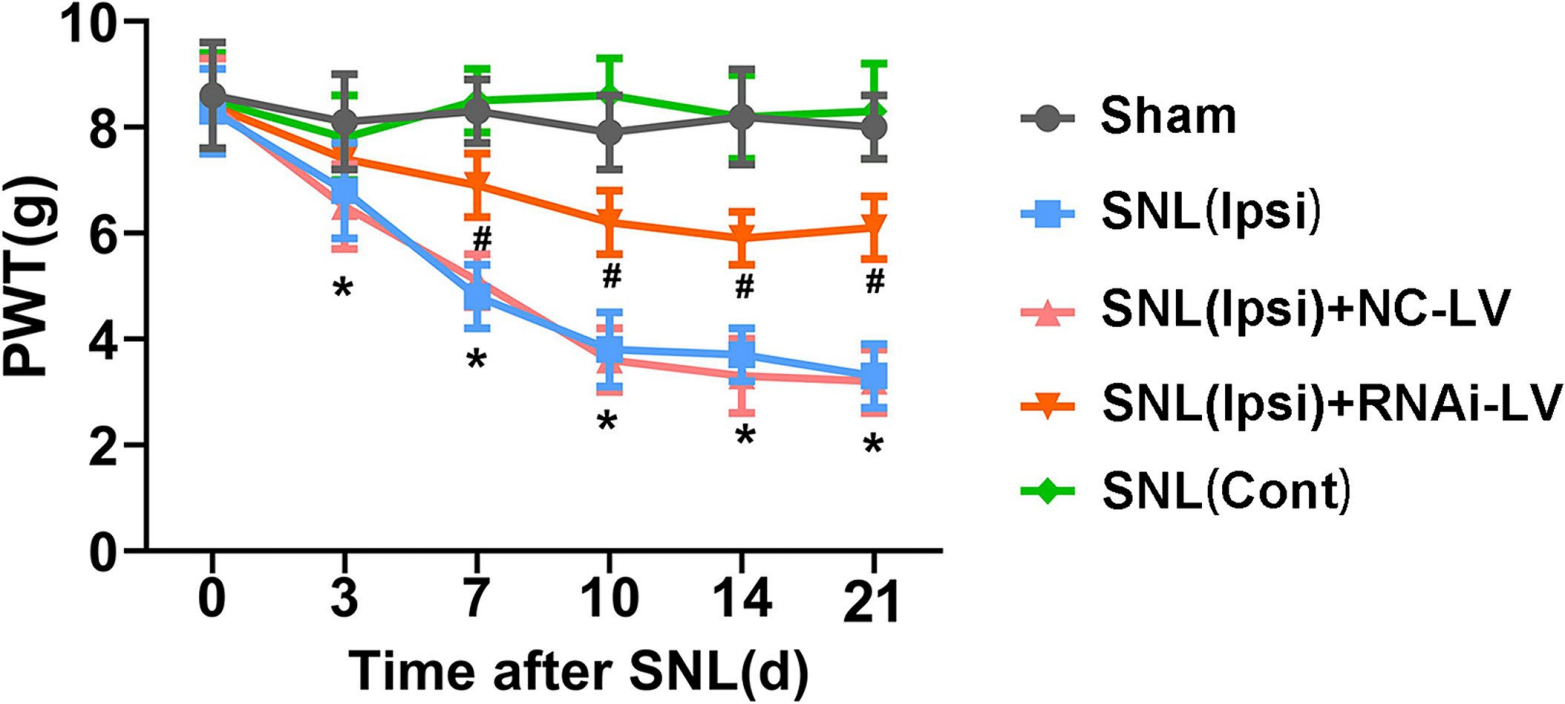
The effect of Dock4 RNAi-LV by spinal microinjection on the development of spinal nerve ligation (SNL)-induced NP. Mechanical allodynia was measured with paw withdrawal threshold (PWT). Pain behaviors induced by SNL were reversed by Dock4 RNAi-LV treatment. *n* = 10 mice/group. *p < 0.05, vs. the sham group; #*p* < 0.05, vs. the SNL + NC-LV group, based on two-way ANOVA, followed by the *post hoc* Tukey test.

**Figure 3:**
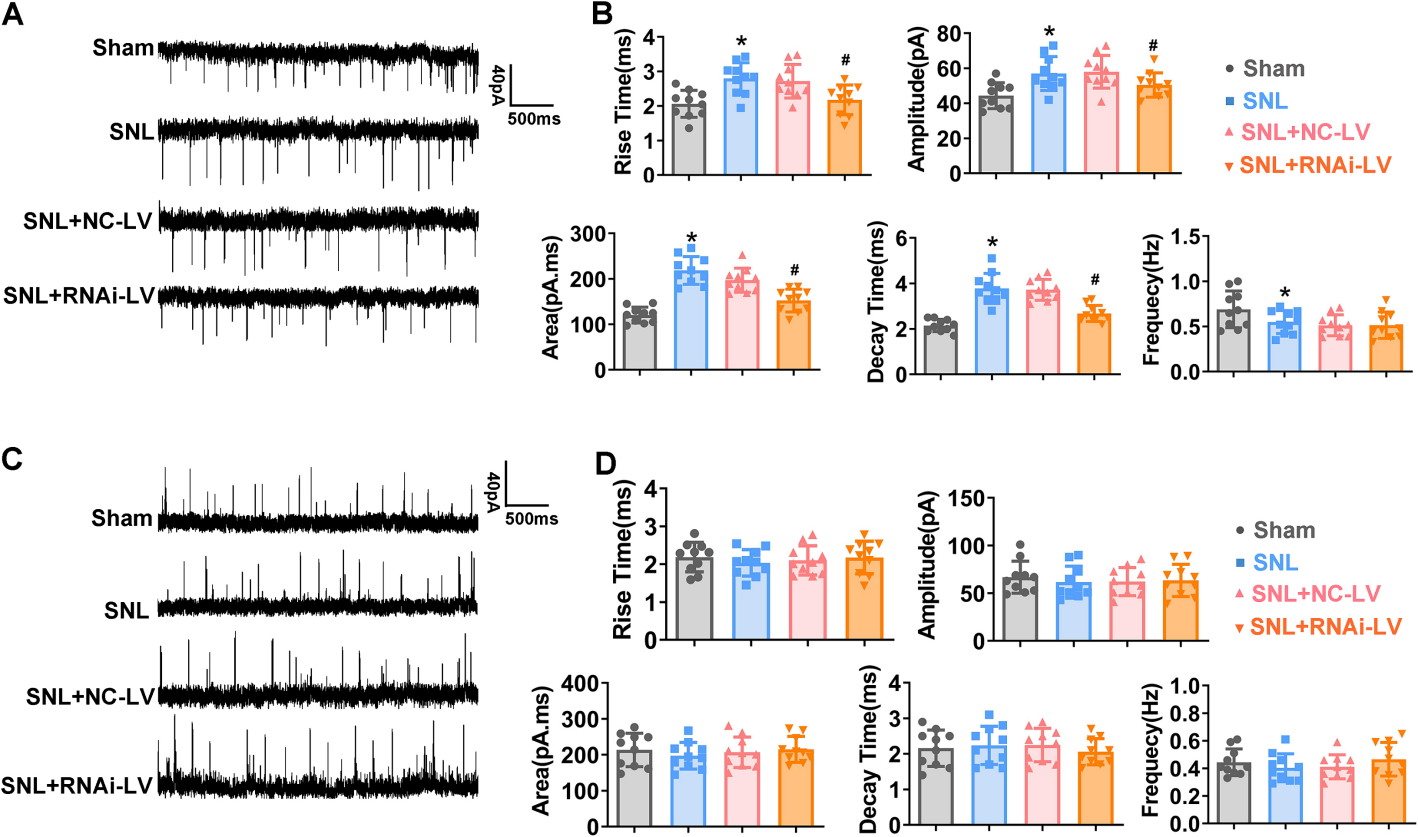
The effects of Dock4 on spontaneous excitatory synaptic currents (sEPSCs) and spontaneous inhibitory post synaptic currents (sIPSCs) in mice. **(A)** Sample traces of sEPSCs recorded in spinal cord slices. **(B)** Rise time, amplitude, area, decay time and frequency of sEPSCs in sham, SNL, SNL + NC-LV, and SNL + RNAi-LV mice. *p < 0.05, vs. the sham group, *n* = 5; #*p* < 0.05, vs. the NC-LV group, by one-way ANOVA and Tukey post-hoc tests. **(C)** Sample traces of sIPSCs recorded in spinal cord slices. **(D)** Rise time, amplitude, area, decay time and frequency of sIPSCs in sham, SNL, SNL + NC-LV, and SNL + RNAi-LV mice. *p < 0.05, vs. the sham group, *n* = 5; #*p* < 0.05, vs. the NC-LV group, by one-way ANOVA and Tukey post-hoc tests.

To explore the hypothesis that the heightened expression of Dock4 in the spinal dorsal horn plays a crucial role in the genesis of NP, we undertook an investigation into the effects of targeted Dock4 suppression within the ipsilateral dorsal horn. This was achieved through microinjection of Dock4- RNAi-LV. Two weeks prior to SNL or sham operations, mice underwent spinal microinjections with either Dock4 RNAi-LV or NC-LV (depicted in Supplementary Figure 1). Subsequent behavioral analyses were conducted at specific intervals post-surgery-days 3, 7, 10, 14, and 21 (referenced in Figure 2). Our findings revealed that SNL elicited a pronounced decline in PWT in response to mechanical stimuli on the ipsilateral side, manifesting by day 3 post-SNL and persisting for the duration of 21 days (depicted in Figure 3C). Remarkably, this heightened sensitivity to pain was substantially mitigated by Dock4 RNAi-LV treatment, an effect that was absent when subjects received NC-LV. Collectively, these results lend strong support to the notion that Dock4 is indispensable for the evolution of neuropathic pain.”

### 3.3 Enhanced Dock4 expression amplifies spinal cord synaptic transmission

To elucidate the synaptic mechanisms through which Dock4 elicits NP, we conducted recordings of spontaneous EPSCs (sEPSCs) and spontaneous IPSCs (sIPSC) in lamina II neurons of sham, SNL, SNL + NC-LV, and SNL + RNAi-LV mice. Our data revealed that SNL precipitated a significant elevation in the rise time, decay time, area, and amplitude of sEPSCs (Figures 3A,B; *p < 0.05). Interestingly, this augmentation was thwarted by the downregulation of Dock4 (Figures 3A,B; #*p* < 0.05). Intriguingly, SNL caused a decline in the frequency of sEPSCs than that in sham mice. However, Dock4 inhibition via RNAi-LV did not affect the altered frequency of sEPSCs following SNL.

It is worth noting that there were no statistically significant disparities in the amplitude and frequency of sIPSCs between sham and SNL groups. Additionally, the knockdown of Dock4 exerted no discernible influence on inhibitory synaptic transmission (Figures 3C,D). To further investigate Dock4’s influence on NMDAR-mediated EPSCs, we proceeded to examine the ratio of NMDAR-EPSCs to AMPAR-EPSCs. Our findings unveiled that the ratio of NMDAR- EPSCs to AMPAR- EPSCs was markedly diminished in spinal dorsal horn neurons extracted from SNL mice followed by Dock4 RNAi-LV treatment, as compared to those treated with NC-LV (#*p* < 0.05). Furthermore, we probed into the functional composition of NMDAR subunits by selectively isolating NMDAR-EPSCs that were inhibited by the GluN2B antagonist ifenprodil (3 μM). Intriguingly, the degree of blockage of NMDAR-EPSCs by ifenprodil was notably less pronounced in spinal dorsal horn neurons from SNL + RNAi-LV mice than in their counterparts treated with NC-LV. These collective data imply that Dock4 participated in NP by enhancing GluN2B mediated excitatory synaptic transmission (Supplementary Figure 2).

### 3.4 Dock4 promotes GluN2B synaptic expression and localization in a Rac1-dependent manner

**Figure 4:**
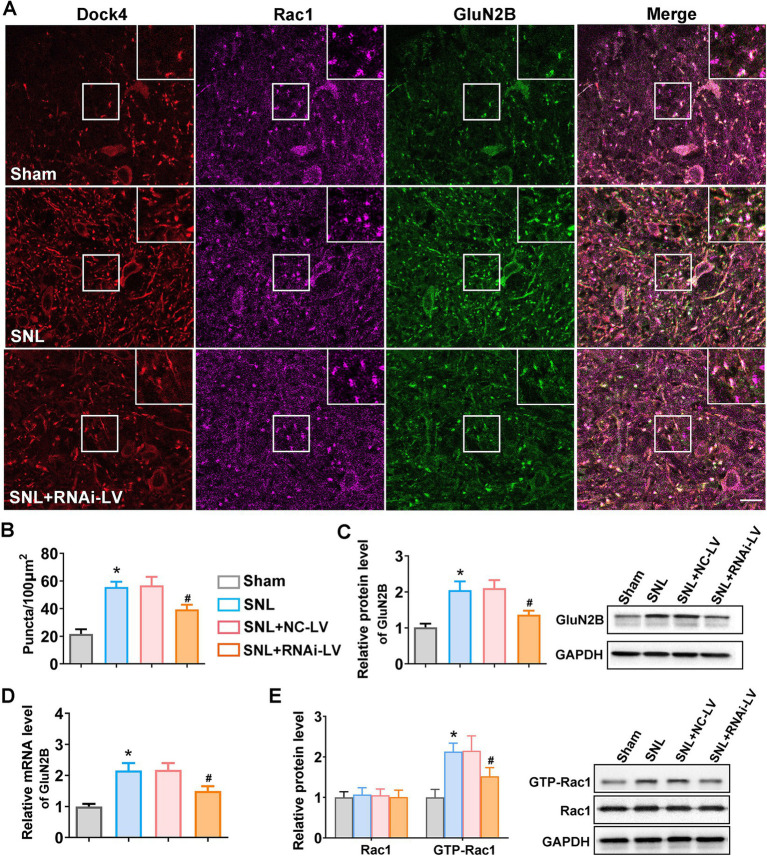
Dock4 facilitates GluN2B synaptic expression and localization in a Rac1-dependent mechanism. **(A)** Representative confocal images of triple immunofluorescent staining of spinal cord slices with anti-Dock4 (purple), anti-Rac1 (red), and anti-GluN2B (green) antibodies. Scale bar: 10 μm. Magnified images of the regions highlighting with white rectangles were shown on the upper right corner. **(B)** Quantitative analysis of the colocalized puncta of Dock4, Rac1 and GluN2B in sham, SNL, and SNL + RNAi-LV mice. Five regions (the number of puncta/100μm^2^) of each slice were analyzed using image J software to quantify the puncta. *p < 0.05, vs. the sham group, *n* = 5; #*p* < 0.05, vs. the NC-LV group. **(C,D)** Quantitative analysis of GluN2B expression using RT-PCR and western blot (*n* = 5). Representative western blots sample of western blot were shown on the right. *p < 0.05, vs. the sham group, *n* = 5; #*p* < 0.05, vs. the NC-LV group. **(E)** Quantitative analysis of Rac1 and GTP-Rac1 expression measured by western blot in sham, SNL, SNL + NC-LV, and SNL + RNAi-LV mice. *p < 0.05, vs. the sham group, *n* = 5; #*p* < 0.05, vs. the NC-LV group.

Emerging evidence has underscored the pivotal role of GluN2B in amplifying nociceptive synaptic transmission within the spinal dorsal horn neurons associated with pain processing. Concurrently, deficiencies in Dock4, coupled with reduced GluN2B expression, have been implicated in eliciting autism-spectrum disorder-like social impairments. Given Dock4’s established function as a GEF for Rac1, we sought to elucidate whether Dock4 exerts its influence over GluN2B receptor synaptic expression through the activation of Rac1. Triple immunofluorescence staining with anti-Dock4, anti-Rac1, and GluN2B antibodies was applied on spinal cord slices. The resulting immunofluorescent images revealed that Dock4, Rac1, and GluN2B were predominantly co-localized at synaptic sites along the neuronal dendrites (Figure 4A). Subsequent quantitative analysis of the co-localized puncta of Dock4, Rac1, and GluN2B confirmed that SNL precipitated an elevation in co-localization, whereas the depletion of Dock4 attenuated this effect (Figure 4B). Complementary quantitative assessments utilizing RT-PCR and western blotting further corroborated these findings. Specifically, SNL was found to upregulate GluN2B expression, an increase that was counteracted by Dock4 knockdown (Figures 4C,D). To delineate the intricate interplay between Dock4 and Rac1, we scrutinized Rac1 expression alongside its active conformation (GTP-bound Rac1) in the presence and absence of Dock4 RNA interference lentivirus (RNAi-LV). Our analyses disclosed that GTP-bound Rac1 levels were markedly elevated in SNL mice; however, this upsurge was abrogated by Dock4 knockdown (Figure 4E). These data suggested that Dock4 exerts a potent facilitative effect on the synaptic expression and localization of GluN2B, executing this influence through a mechanism fundamentally anchored in Rac1 activation.

### 3.5 Dock4 governs dendritic spine remodeling and synaptogenesis in spinal dorsal horn neurons

**Figure 5:**
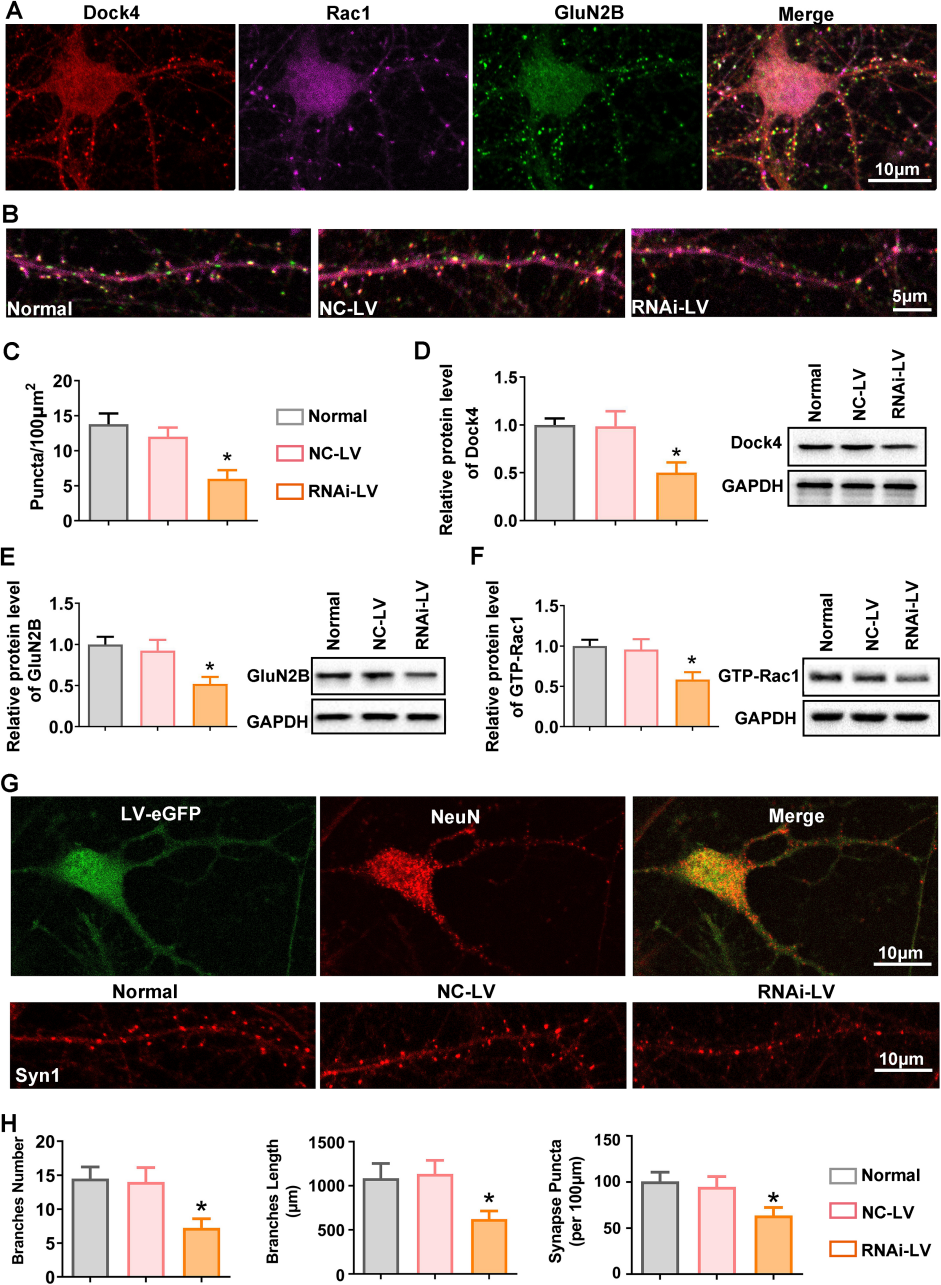
Dock4 orchestrates dendritic spine remodeling and synaptogenesis in spinal dorsal horn neurons. **(A)** Representative images of immunofluorescence triple staining of Dock4 (red), Rac1 (purple), and GluN2B (green) in cultured spinal dorsal horn neurons. **(B,C)** Quantification analysis of Dock4, Rac1 and GluN2B puncta along dendrites in untreated neurons, neurons treated with negative control lentivirus (NC-LV), and neurons treated with Dock4-specific RNAi lentivirus (RNAi-LV). *n* = 5, *p < 0.05, vs. the NC-LV group. **(D–F)** Quantification analysis of Dock4, Rac1 and GluN2B expression in untreated neurons, NC-LV-treated neurons, and RNAi-LV-treated neurons, assessed by western blotting. A representative western blot is shown on the right. *n* = 5, *p < 0.05, vs. the NC-LV group. **(G)** Immunostaining for NeuN highlights neuronal branches, while immunostaining for Syn1 reveals synaptic puncta. Lentivirus-transfected neurons are visualized by green fluorescence. **(H)** Quantification analysis of branch number and length and Syn1 puncta along branches in untreated neurons, NC-LV-treated neurons, and RNAi-LV-treated neurons. *p < 0.05, vs. the NC-LV group. Data are presented as means ± SEM, one-way ANOVA followed by Tukey’s *post hoc* test.

Synaptic structural remodeling and functional alterations are intrinsically intertwined processes. A plethora of research has underscored the pivotal role of endogenous GluN2B in promoting dendritic growth (Sceniak et al., 2019; Gonda et al., 2020; Bahry et al., 2021). Given that Dock4 exhibits widespread expression across neurons in the spinal cord and exerts a regulatory influence on GluN2B expression *in vivo*, we sought to extend our investigative efforts to primary cultured dorsal spinal neurons. This approach was undertaken with the primary objective of delineating the precise contributions of Dock4 to the intricate process of synaptogenesis. Triple immunofluorescence staining was conducted to assess the expression and co-localization of anti-Dock4, anti-Rac1, and GluN2B (Figure 5A). As depicted in Figures 5B,C, juxtaposed against NC-LV group, the transfection of neurons with Dock4 RNAi-LV precipitated a discernible diminution in the number of co-localized puncta. To corroborate the pivotal role of Dock4 in modulating Rac1 and GluN2B expression, we conducted western blot analysis on neurons transfected with lentivirus. Consistent with our *in vivo* observations, the knockdown of Dock4 was found to attenuate the expression of Dock4, GTP-bound Rac1, and GluN2B (Figures 5D–F), thereby reinforcing the interdependence of these molecular entities. To further probe the impact of Dock4 on synaptogenesis, we quantified synaptic development through immunofluorescence staining targeting synapsin-1, a marker indicative of synaptic sites. Our quantitative analysis revealed that the introduction of Dock4 RNAi-LV led to a significant decline in dendritic spine density, branch number, and length (Figure 5G). This finding underscores the critical role of Dock4 in the maintenance and refinement of synaptic architecture, suggesting its potential as a regulator of synaptic plasticity and neuronal connectivity.

## 4 Discussion

In the present investigation, we unveiled that SNL instigates an upregulation of Dock4 within spinal dorsal horn in murine models. This elevation in Dock4 expression potentiates excitatory synaptic transmission and facilitates the synaptic expression and localization of GluN2B, operating through a mechanism critically reliant on Rac1 activation. Spinal microinjection of Dock4 RNAi-LV mitigated the hypersensitivity to pain triggered by SNL, concurrently downregulating excitatory synaptic transmission and curtailing the augmented expression of GTP-bound Rac1 and GluN2B. Furthermore, our *in vitro* experiments elucidated that Dock4 governs dendritic spine remodeling and synaptogenesis in neurons originating from the spinal dorsal horn. Collectively, these findings delineate a previously unacknowledged mechanism by which Dock4, in concert with Rac1, regulates the functionality of GluN2B at the spinal level, thereby exerting an influence on NP.

Impaired synaptic plasticity within the pain circuitry of the spinal dorsal horn constitutes a cardinal mechanism underlying the genesis of NP. The remodeling of synaptic structures, cellular constituents, and neural circuits, particularly the reconfiguration of dendritic spines, precipitates prolonged chronic pain states. Prior literature has attested to the modulation of dendritic spine formation and architecture by a myriad of signaling cascades, chiefly through the regulation of the actin cytoskeleton (Okabe, 2020; Suratkal et al., 2021; Tu et al., 2023). Research has elucidated the pivotal role of the Rac1 signaling pathway in augmenting dendritic spine density in the spinal dorsal horn, thereby implicating it in the pathophysiology of chronic pain (Wu et al., 2023; Xu et al., 2023). As a Rac1-GEF that couple synaptic receptors with Rac1 signaling, Dock4 is highly expressed in the spinal cord, developing brain, and adult brain. Recent studies have revealed the important role of Dock4 in autism-like social deficit (Guo et al., 2021). Our findings herein corroborate that Dock4 predominantly localizes within neurons of the spinal dorsal horn, mirroring its distribution profile in hippocampal neurons as delineated in previous reports. The marked upsurge in Dock4 expression post-SNL underscores the transcriptional activation of the Dock4 gene during the onset of NP. Spinal microinjection of Dock4 RNAi-LV can effectively inhibit SNL- induced mechanical allodynia, and hyperalgesia. In an *in vitro* setting, Dock4 RNAi-LV transfection resulted in a decrement in dendritic spine density. These results indicated that Dock4 could control dendritic spine remodeling and participate in the occurrence of NP.

NMDARs are integral to a spectrum of physiological and pathological phenomena, encompassing neurodevelopment, excitatory synaptic transmission of neurons, synaptic plasticity, central sensitization, and neuronal death (Paoletti et al., 2013; Ge et al., 2020; Zong et al., 2022). It plays a crucial role in the mechanisms of peripheral and central sensitization (Liu et al., 2022; Xie et al., 2022; Han et al., 2024). The expression changes of GluN2B are the main mechanism regulating the plasticity alterations of NMDARs (Popov et al., 2020; Crivellaro et al., 2021). Several lines of evidence showed that GluN2B was upregulated in the spinal dorsal horn and dorsal root ganglion after peripheral nerve injury (Jovanovic et al., 2020; Lenoir et al., 2020; Nimbalkar et al., 2023), and yet the upstream regulatory mechanism of GluN2B remain unclear. Provide evidence that GluN2B could be regulated and activated by Rac1 underlying NP. In this study, we found that activation of Dock4 increased the membrane expression of GluN2B. Knockdown of Dock4 prevented SNL-induced upregulation of GluN2B and mechanical allodynia. These results indicated that Dock4 is fundamental to traffic and anchor GluN2B at synaptic sites. Our data suggested that Dock4 mediates regulation of NP in a Rac1- GluN2B manner.

The increase in the release of presynaptic neurotransmitters or the hyperactivity of postsynaptic membrane glutamate receptors may both lead to the enhancement of synaptic transmission (Magee and Grienberger, 2020; Platholi and Hemmings, 2022; Yan and Rein, 2022). Since Dock4 modulated the expression of GluN2B, we further investigated the role of Dock4 in excitatory synaptic transmission in NP. Electrophysiology data indicated that knockdown of Dock4 inhibited SNL-induced upregulation of the rise time, decay time, area, and amplitude of sEPSCs, yet it has no impact on sEPSC frequency in spinal dorsal horn neurons. These results indicated Dock4 participated in NP by enhancing GluN2B mediated excitatory synaptic transmission.

Nonetheless, within the scope of our investigation, the application of RNA interference (RNAi) directed against Dock4 manifests limited effectiveness in antagonizing the upregulation of Rac1 and GluN2B protein expression induced by SNL. Similarly, the suppression of augmented sEPSCs is comparably restrained. This limited effectiveness could stem from several factors. Firstly, the RNAi strategy itself may possess inherent limitations in achieving complete silencing of Dock4, potentially due to suboptimal target site selection, off-target effects, or incomplete knockdown efficiency. Secondly, these findings might hint at the involvement of additional, Dock4-independent pathways or compensatory mechanisms that contribute to the observed alterations in Rac1, GluN2B expression, and sEPSC activity. Therefore, while Dock4 plays a significant role in these processes, it is likely not the sole mediator, suggesting a complex interplay of multiple factors in the pathophysiology of neuropathic pain following SNL. Additional investigations are warranted to elucidate the precise mechanisms underpinning these observations.

Taken together, our study explored the vital role of Dock4 in the development of NP, which could provide new targeted drugs for the treatment and relief of NP.

## Data availability statement

The original contributions presented in the study are included in the article/ Supplementary material, further inquiries can be directed to the corresponding author.

## Ethics statement

The animal study was approved by Animal Use and Care Committee of Hubei University of Medicine. The study was conducted in accordance with the local legislation and institutional requirements.

## Author contributions

QF: Funding acquisition, Project administration, Writing – original draft. HL: Project administration, Writing – original draft. ZZ: Data curation, Investigation, Methodology, Writing – review & editing. WL: Formal analysis, Software, Writing – review & editing. ZR: Resources, Validation, Writing – review & editing. RC: Validation, Visualization, Writing – review & editing. HW: Formal analysis, Resources, Validation, Writing – review & editing. XueX: Data curation, Methodology, Writing – review & editing. XunX: Data curation, Writing – review & editing, Formal analysis, Funding acquisition. YW: Funding acquisition, Supervision, Validation, Writing – review & editing.
