# Supplementary material for: Dock4 contributes to neuropathic pain by regulating spinal synaptic plasticity in mice
Source: Front Mol Neurosci. 2024 Aug 30;17:1417567. doi: 10.3389/fnmol.2024.1417567 (PMC11392915; doi:10.3389/fnmol.2024.1417567)
Supplement: Supplementary file 1 [file Table_1.docx]

**Supplementary data**


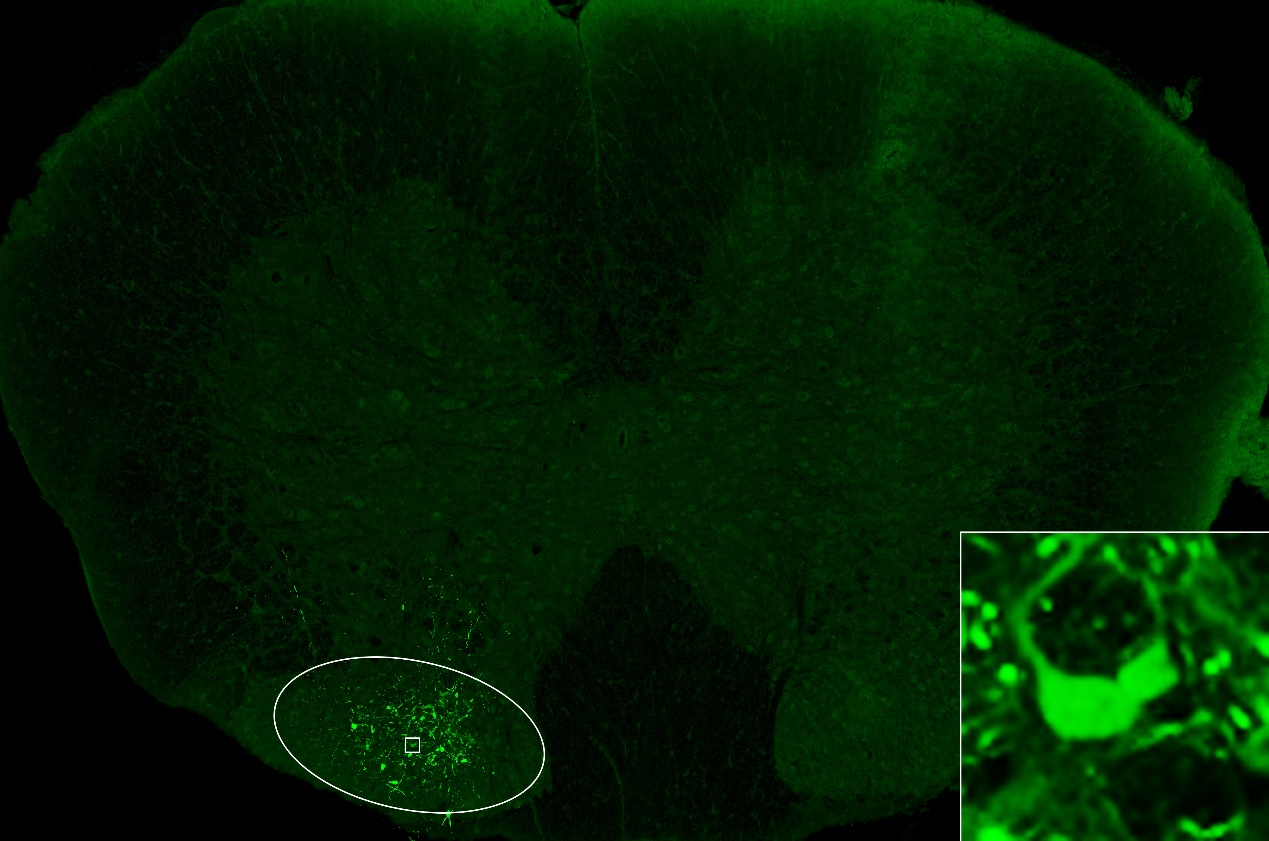


Supplementary figure 1 A spinal cord cross-section with the area of virus-transfection outlined (White ellipse). The internal white box is a partial enlarged view


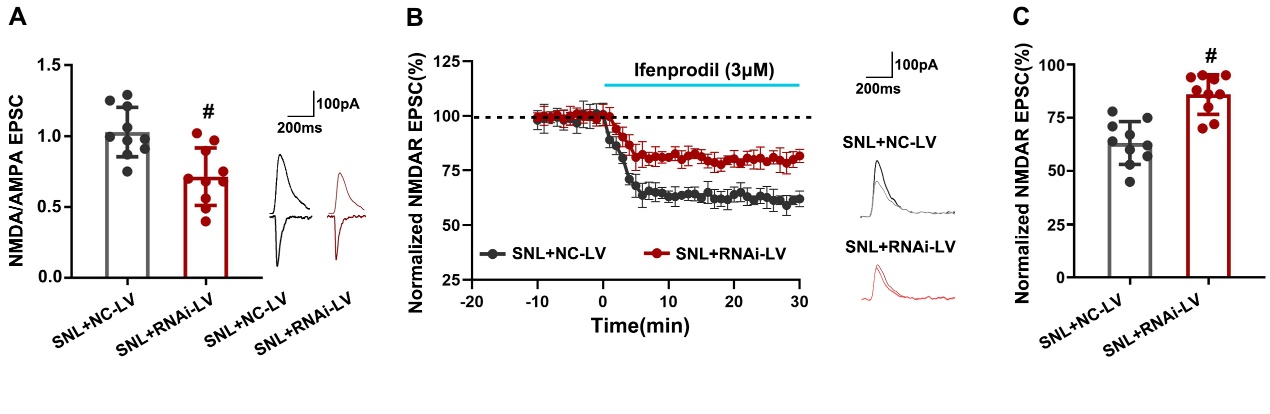
Supplementary Figure 2 The AMPAR and NMDAR-dependent synaptic transmission of spinal dorsal horn neurons in mice. (A) The NMDAR/AMPAR ratio of EPSCs was decreased in spinal dorsal horn neurons from SNL+RNAi-LV mice, compared with NC-LV mice (#*p*<0.05 compared with NC-LV, unpaired t-test). n = 10 cells from 5 mice/group; Representative traces are shown on the right. (B) Time-course changes of NMDAR-EPSC (% of baseline) after application of ifenprodil, an GluN2B inhibitor. Representative traces before and at 20–30 min after application of ifenprodil are shown on the right. n = 10 cells from 5 mice/group. (C) SNL+RNAi-LV mice showed a reduction in the proportion of synaptic GluN2B. NMDAR-EPSC (% of baseline) were analyzed 30 minutes following the administration of ifenprodil (#*p*<0.05 compared with NC-LV, unpaired t-test); n = 10 cells from 5 mice/group.
